# Post-Traumatic Growth among Patients after Living and Cadaveric Donor Kidney Transplantation: The Role of Resilience and Alexithymia

**DOI:** 10.3390/ijerph18042164

**Published:** 2021-02-23

**Authors:** Aleksandra Tomaszek, Aleksandra Wróblewska, Elżbieta Zdankiewicz-Ścigała, Patryk Rzońca, Robert Gałązkowski, Jolanta Gozdowska, Dorota Lewandowska, Dariusz Kosson, Maciej Kosieradzki, Roman Danielewicz

**Affiliations:** 1 Teaching Department of Anaesthesiology and Intensive Care, Faculty of Health Sciences, Medical University of Warsaw, 02-091 Warsaw, Poland; aleksandra.koordynacja@onet.pl (A.T.); kosson@wp.pl (D.K.); 2 Faculty of Psychology, SWPS University of Social Sciences and Humanities, 03-815 Warsaw, Poland; wroblewska.aleksandra.anna@gmail.com (A.W.); ezdankiewicz-scigala@swps.edu.pl (E.Z.-Ś.); 3 Department of Emergency Medical Services, Faculty of Health Science, Medical University of Warsaw, 02-091 Warsaw, Poland; r.galazkowski@lpr.com.pl; 4 Department of Transplant Medicine, Nephrology and Internal Diseases, Medical University of Warsaw, 02-091 Warsaw, Poland; jola-md@o2.pl (J.G.); dlewandowska@poltransplant.pl (D.L.); 5 Department of General and Transplant Surgery, Medical University of Warsaw, 02-091 Warsaw, Poland; mpkosieradzki@gmail.com (M.K.); rdanielewicz@poltransplant.pl (R.D.)

**Keywords:** kidney transplantation, psychology, resilience, alexithymia, post-traumatic growth

## Abstract

The aim of this study was to determine the role of resilience and alexithymia in the post-traumatic growth as a response to extreme stress in patients after kidney transplantation and to determine whether there are differences in the level of posttraumatic growth in patients after living and cadaveric donor kidney transplantation. The relationships between these variables were also evaluated. The questionnaire survey of 91 kidney recipients took place in 2018 and 2019. The following tools were used: authorial post-transplant questionnaire for recipients and validated questionnaires, Post Traumatic Growth Inventory (PTGI-R), Resilience Coping Scale Questionnaire, and Toronto Alexithymia Scale Questionnaire (TAS20). The results obtained showed significant differences between the group of kidney recipients from living donors and recipients from cadaveric donors, in terms of overall post-traumatic growth, as well as changes in self-perception and a greater appreciation for life. Post-traumatic growth in both groups was related to the level of resilience and the level of alexithymia. Resilience is an accurate predictor of posttraumatic growth in general and for each of the groups of recipients separately.

## 1. Introduction

Organ transplantation is the removal of an organ from one person (the donor) and placing it into the body of another person (the recipient). Transplantation is performed in patients with end-stage organ failure. It can extend the life expectancy while improving its quality. The kidney is the most commonly transplanted solid organ, followed by the liver, heart, and lungs. Organs can be retrieved from living (kidney or part of the liver) or cadaveric (all organs) donors, but cadaveric donors are more common [1].

Kidney transplantation is a surgical procedure performed in patients with end-stage renal disease [2,3]. In Poland, kidneys for transplantation are most often harvested from donors who died in the mechanism of brain death. Mostly, they were people aged 40–60, without significant medical burdens, who suffered from a hemorrhagic or ischemic stroke of the central nervous system and then the brain death [4]. Studies on the psychological consequences of kidney transplantation report that the process of kidney transplantation from living donor is an experience that evokes strong emotions for donors [5]. The intensity of these emotions changes in the process of qualifying for a transplant. There are patients for whom kidney donation is a traumatic experience. We noted a higher rate of depressive disorders in donors as compared to the control group. There were also changes in mental functioning and differences in the quality of life. The process may be accompanied by a developmental or existential crisis. On the other hand, Tomaszek et al. [6] report that in the event of extreme stress, such as kidney transplantation, the level of mental resilience in kidney donors increases as compared to the level before the procedure. These results are consistent with the theory that resilience emerges at the times of danger and that it can be used to help to cope with the disease. Following the kidney donation, the study patients were more able to accept the difficult situation and to deal with the emotions that would naturally arise at such times [6].

The basis for the concept of post-traumatic growth are the positive psychological changes accompanying highly stressful events. Variables such as an individual’s ability to experience benefits, stress-related growth, personality development, transformational coping, and growth resulting from adversity are also used to characterize the post-traumatic growth. The term “post-traumatic growth” was introduced into literature in 1996 by Tedeschi and Calhun [7], who believed that the described phenomenon occurs in conditions of severe crisis but not an ordinary stress, is rather an effect and not a coping mechanism, and goes beyond the illusion of change. It requires a change in basic assumptions about one’s life. Thus, this concept contains a number of positive changes that occur as a result of highly traumatizing events. Individuals undergo strong transformation, empowerment, positive and long-lasting psychological changes, the ability to see or create benefits, and even development. Posttraumatic growth is also referred to as flourishing, transformational coping, discovery of meaning, positive illusion or drawing strength from adversity. The individual can confront problems, the past, and the future [7].

The main mechanism enabling posttraumatic growth is resilience [8,9]. The literature on this topic uses two terms: resilience and resiliency. This first one is referred to as resistance (resilience) and is equated with the process of effectively overcoming negative phenomena and life events. The second understanding means a relatively durable resource of an individual and is referred to as mental resilience [10]. Block and Kremen [11] described mental resilience as a personality attribute, important in the process of struggling both with traumatic events and events occurring in everyday life. Resilience is in opposition to the lack of control or to its excess and is associated with the individual’s ability to adapt the level of control to his or her own abilities and situations. It has been proven that the level of resilience increases with openness to new experiences, a sense of humour, personal competence related to toleration of negative emotions, an optimistic approach to life and the ability to mobilize [11].

When an individual experiences a traumatic event, alexithymia plays the role of a defense mechanism [12]. The inability to find the right words to describe one’s own emotional states is the deepest root of alexithymia [13]. Alexithymics cannot modulate their emotional processes using cognitive processes [14]. Alexithymia is associated with other disorders, such as Post-Traumatic Stress Disorder (PTSD), mood and anxiety disorders, and substance abuse [15].

The aim of the study was to determine the role of resilience and alexithymia in the occurrence of posttraumatic growth as a reaction to extreme stress in people after kidney transplantation and to determine whether there are differences in the level of posttraumatic growth in people after kidney transplantation from living and cadaveric donors. The relationships between these variables were also evaluated.

## 2. Materials and Methods

### 2.1. Participants

The study was conducted from November 2018 to April 2019 in patients who underwent kidney transplantation in the Department of General and Transplant Surgery and Department of Transplantation Medicine, Nephrology and Internal Diseases of the Medical University of Warsaw, University Clinical Center, the Infant Jesus Clinical Hospital in Warsaw, Poland. The inclusion criteria for participation in the study were: recipient of kidney from cadaveric or living donor, age 18 or more, and informed consent. There were 91 kidney transplant recipients recruited and participated in the study, including 44 recipients of kidneys from living donors, and 47 recipients from cadaveric donors. The respondents were women and men, aged 18 to 73, Poles living in various regions of the country. All procedures in this study followed the principles of Helsinki Declaration, the APA ethical standards (Including 2010 and 2016 Amendments) and were approved by the Bioethics Committee of the Medical University of Warsaw (no. AKBE/130/17). The study was, moreover, approved by the managers of the hospital departments involved. The patients taking part in the project were informed about the voluntary nature of their participation, their anonymity, and the fact that the results of the study would be used exclusively for scientific purposes. Before filling up the questionnaires, the participants were asked to sign an informed consent form which specified all their tasks and rights.

### 2.2. Assessment

The study used a diagnostic survey with questionnaires. The following tools were used to conduct the study: self-prepared post-transplant questionnaire for recipients, Post Traumatic Growth Inventory PTGI-R Questionnaire (PTGI-R), Resilience Coping Scale Questionnaire (SPP25), and Toronto Alexithymia Scale Questionnaire (TAS20).

The authorial post-transplant questionnaire for recipients was designed to collect information on gender, age, place of residence, life situation, type of donor (living or cadaveric), type of relationship with a donor (in the case of living donors), assessment of psychological costs, psychological and health status, health care and assessment of changes in the quality of life and support from relatives. The respondents estimated the above-mentioned variables on a five- and seven-point scale.

The PTGI-R questionnaire was developed by Tedeschi and Calhoun, adapted to the Polish language by Ogińska-Bulik and Juczyński [7,16]. The questionnaire was intended to determine the type of traumatic event that changed life and to describe the degree of change experienced as a result of the crisis. The degree of change was determined in 21 points, and a scale from 0 (I did not experience this change as a result of my crisis) to 5 (I experienced this change to a very great degree as a result of my crisis) was used for the description. The Polish adaptation of the tool measured 4 factors related to the development of trauma: self-perception, changes in relationships with others, greater appreciation of life and spiritual changes. The scoring was assigned positively to the intensity of positive changes. The Polish adaptation of the PTGI-R questionnaire is characterized by good internal consistency (Cronbach’s alpha = 0.93) and high reliability measured by a test-retest at a 2-month interval (r = 0.74) [16].

The TAS20 questionnaire was developed by Taylor, Bagby and Parker [17] and measured the occurrence or absence of alexithymia. The TAS-20 questionnaire was developed as a result of a revision of the alexithymia self-assessment scale, TAS-26, which has been reduced to 20 items. TAS20 assesses 3 factors: difficulties in identifying feelings, difficulties in describing feelings and tendency to operational thinking, and is the most frequently used scale to assess alexithymia. For each item, a 5-point Likert scale was used, with the range from 1-I disagree very much to 5-I agree very much. When interpreting the results of the TAS20 scale, the value of 56 points for alexithymia and the value of 44 points for the absence of alexithymia features were adopted in accordance with other alexithymia studies. The TAS-20 scale is characterized by good internal consistency (Cronbach’s alpha = 0.81) and high reliability as measured by a 3-week test-retest (r = 0.77) [17,18,19,20].

The Resilience Coping Scale, developed by Ogińska-Bulik and Juczyński [10], in 25 points on a scale from 0 (definitely not) to 5 (definitely yes), checked how the respondent behaved in difficult situations. The adaptation of foreign-language versions was abandoned due to the multiplicity of methods and the lack of a clear preference for a specific tool. A scale was constructed to measure resilience, treated as a personality trait important in the process of effectively coping with both traumatic events and everyday stress. Internal compliance, based on Cronbach’s alpha, is 0.89 for the whole scale, and high reliability was measured by a 4-week test-retest (r = 0.85) [10].

### 2.3. Statistical Analysis

The results were statistically analyzed using the IBM SPSS Statistics 25.0 software (IBM Corporation, Armonk, NY, USA). The normality of variable distribution was performed using the Shapiro–Wilk normality test. For the assessment of differences in the level of post-traumatic growth between recipients from a living donor and recipients from a cadaveric donor, parametric t-Student test for two independent samples was used. Spearman’s rho and Pearson’s rho correlation coefficient (corresponding to the distribution of variables) between resilience, alexithymia and post-traumatic growth were used. In order to examine the relationships between variables (resilience and post-traumatic growth), one-variable linear regression analysis was used. The study assumed statistical significance as *p* < 0.05.

## 3. Results

The study included 91 patients, women (47%) and men (53%). Their age ranged from 18 to 73 years old, on average 42 (SD = 13). 44 patients (48%) received kidney from living donor, 47 patients (52%) from cadaveric donor. The average time since transplantation was 8 years (ranged from 1 year to 27 years).

The results showed significant differences between the group of recipients from a living donor and the group of recipients from a cadaveric donor in terms of overall post-traumatic growth t (86,204) = 2.271; *p* = 0.03, as well as changes in self-perception t (89) = 2.189; *p* = 0.03 and greater appreciation of life t (82,247) = 2.524; *p* = 0.01. There were no differences in the case of changes in relationships with others or spiritual changes (*p* > 0.05) (Table 1).

The average score for overall post-traumatic growth was higher for recipients from a living donor (M = 74, SD = 16) than from a cadaveric donor (M = 65, SD = 21). The average score for changes in self-perception was also higher for recipients from a living donor (M = 31, SD = 9) than to recipients from the cadaveric donor (M = 27, SD = 11). Similarly, the average score for life appreciation was higher for recipients from a living donor (M = 13, SD = 2) than for a cadaveric donor (M = 11, SD = 4) (Table 1).

The analysis for living donor kidney recipient showed that post-traumatic growth (overall result, change in self-perception, and change in relationships with others) correlate positively with resilience (in general and with all its factors, except tenacity and determination). Additionally, greater appreciation of life correlates positively with resilience (in general) and with personal competencies of dealing with and tolerating negative emotions, tolerance for failure and approaching life as a challenge, as well as optimistic approach to life and the ability to mobilize. Spiritual changes correlate with tenacity and determination. The results indicate that the level of post-traumatic growth increases with the increase in the level of resilience. The most important factor among all the factors of resilience is failure tolerance and approaching life as a challenge. The least important factor is tenacity and determination. Changes in the level of resilience is of the least importance to spiritual changes (Table 2).

The analysis for cadaveric donor kidney recipients showed that posttraumatic growth (overall result, change in self-perception, and a greater appreciation for life) correlate positively with resilience (in general) and with all its factors. Additionally, changes in relationships with others positively correlate with resilience (in general) and with tenacity and determination. There was no correlation between resilience and spiritual change. These results also indicate that the level of post-traumatic growth increases with the increase in the level of resilience. The most important factor among all the factors of resilience for this group of patients is tenacity and determination (which was the least important in the group of living donor kidney recipients). The least important factor is openness for new experiences and sense of humor. Changes in the level of resilience is not important to the level of spiritual changes (Table 3).

Table 4 and Table 5 shows the values of the correlation coefficient of alexithymia with post-traumatic growth in a recipient from a living and cadaveric donor. The presented results showed that in living donor kidney recipients only spiritual changes correlate positively, moderately with outward-orientated thinking. There was no correlation between the other variables. The results indicate that the level of one factor of post-traumatic growth (spiritual changes) increases with the increase in the level of one factor of alexithymia (outward-orientated thinking).

The level of alexithymia (overall result) correlated with post-traumatic growth (overall result) only in recipients from a cadaveric donor. Posttraumatic growth (overall result), changes in self-perception, and a greater appreciation for life correlated negatively, moderately with alexithymia (in general) and with all its aspects except outward-oriented thinking. No correlations were observed between alexithymia and changes in relationships with others and with spiritual changes. The results indicate that the level of post-traumatic growth decreases with the increase in the level of alexithymia. There was only one factor among all the factors of alexithymia important for the group of recipients from a living donor, which was outward-orientated thinking. It was not important at all for the group of recipients from a cadaveric donor.

We assumed that the level of resilience can be a predictor for post-traumatic growth in all recipients in general and recipients from a living donor and recipients from a cadaveric donor separately. To examine the relationship between variables, univariate linear regression analysis was used.

In the case of model common for both examined groups (recipients from living and cadaveric donor), the relationship between the predictor and the dependent variable was moderate and positive (beta = 0.445). The higher the resilience of the subjects, the higher the post-traumatic growth. The value of b1 was 0.557; t (86) = 4.507; *p* < 0.001 and the value of constant b = 27.622; t (86) = 2.968; *p* = 0.004. This means that when the resilience level increases by one unit, the posttraumatic growth level will increase by 0.557 (Table 6).

In the model for the group of living donors, the relationship between the predictor and the dependent variable was also moderate and positive (beta = 0.442). The value of the b1 was 0.529; t (41) = 3.157; *p* = 0.021 and the value of the constant b = 31.737; t (41) = 2.402; *p* = 0.021. This means that when the level of resilience increases by one unit, the level of posttraumatic growth will increase by 0.529 (Table 7).

In the model for the group of cadaveric donors, the relationship between the predictor and the dependent variable was moderate and positive (beta = 0.424). The value of the b1 was 0.543; t (43) = 3.066; *p* = 0.004 and the value of the constant b0 = 26.810; t (43) = 2.014; *p* = 0.050. This means that when the level of resilience increases by one unit, the level of posttraumatic growth will increase by 0.543 (Table 8).

## 4. Discussion

The psychological adaptation of the donor and recipient to the transplantation sometimes triggers intrapsychological conflicts that need to be investigated and recognized in a timely manner, bearing in mind their importance in pre- and post-transplant procedure [3]. The mixture of organic and psychological symptoms is characteristic for psychological changes in transplant patients. Free from the dialysis and intensive contact with the medical staff, patients may feel insecure and unprotected, inundated with an ambivalent attitude to their new abilities and old desires. A kidney transplantation improves the patient’s physiological balance and, on a psychological level, restores confidence [21].

Tomaszek et al. [6] focused in their study on donors by checking if a situation of extreme and traumatizing stress, such as living kidney donation, will result in changes in the quality of their life: whether a posttraumatic growth should occur, and if the living donor develops a strategy to handle strong and uncommon stress, known as resilience. This is why we made an attempt to further investigate the from a living donor and observed a higher post-traumatic growth in general, in terms of changes in self-perception and a greater appreciation for life. The occurrence of post-traumatic growth in response to extreme stress, i.e., kidney transplantation, is consistent with the assumptions presented by Tedeschi and Calhoun [22], that one of the consequences of experiencing a major crisis is post-traumatic growth, which should be considered in terms of the result, not coping mechanisms. The obtained phenomena related to kidney transplantation and to specify the influence of the transplantation procedure on recipient by comparing the group of living donor recipients with those of the cadaveric donors. Kidney recipients were examined in terms of post-traumatic growth, resilience and alexithymia.

In the group of recipients after transplantation results also comply with the findings of the study by Tomaszek et al. [6], who showed that the consequence of kidney transplantation in live kidney donors is the occurrence of posttraumatic growth, which was observed half a year after the surgery. According to the authors, living kidney donation can be compared to giving life, which strongly affects the psyche of both the donor and recipient. Strengthening the relationship between recipient and donor could contribute to the higher post-traumatic growth observed in the present study in recipients who received a kidney from a loved one than in recipients who received it from a deceased person. The impact of a close relationship on the growth phenomenon was confirmed by the reports of Ogińska-Bulik [23], who reported that the course and result of the post-traumatic growth phenomenon was related to the social support experienced by an individual and their personal resources [23].

In this study, the level of resilience was associated with post-traumatic growth in both recipients from a living donor and from a cadaveric donor. The results showed a moderate or strong positive correlation of posttraumatic growth both in general and in its specific aspects. Tomaszek et al. [6] obtained similar results in a study of living kidney donors, finding the strongest correlation of post-traumatic growth with personal resistance to failure and treating life as a challenge, as well as an optimistic approach to life and the ability to mobilize. These results were also confirmed by Ogińska-Bulik and Juczyński [24] in studies conducted on a group of patients with cancer after breast resection. Similar results were presented in the studies of Yongju et al. [25], which confirmed the impact of resilience and social support on the occurrence of posttraumatic growth in women suffering from infertility [25].

The discussed results illustrate that people with high resilience treat life as a challenge, are optimistic and able to mobilize themselves in a difficult situation. It promotes growth and development after extreme stress.

Researchers conducting this study exposed a relationship between posttraumatic growth and alexithymia. In the case of recipients from the cadaveric donor, this was a negative, moderate correlation between post-traumatic growth (overall), changes in self-perception and greater appreciation of life, and all aspects of alexithymia except for outward-oriented thinking. There was no correlation of the overall results for the variables studied. The results of a group of recipients from a living donor find partial confirmation in research, including Orejuel-Dávila et al. [26] on students after surviving extreme stress, in which the correlation of alexithymia with posttraumatic growth was examined. There was no simple correlation, but the relationship between the variables was observed as a result of regression analysis [26].

In this study, we aimed to present the role of psychological conditions, such as resilience and alexithymia on the post-traumatic growth among patients after living and cadaveric donor kidney transplantation as we have not found such studies in the literature so far.

The study group was single-center and relatively small, due to the limited number of living donor kidney transplant recipient in Poland. The results should be verified in larger and multi-center populations.

In the recipient group from the cadaveric donor a higher number of patients with a low level of post-traumatic growth was observed, however, a comparison of categorical variables was impossible due to insufficient sample.

Post-traumatic growth requires time, and presented study was a cross-sectional one. Therefore, in future studies a longitudinal study should be conducted to accurately reflect this dynamic process.

The observed differences between recipients from a living donor and cadaveric donor encourage exploring both the causes and effects of these differences, comparing the subjects also in terms of their somatic health and the organism’s response to the transplanted organ. The effect of psychological impact, including psychotherapy, on both patient groups would also be interesting.

## 5. Conclusions

- Resilience correlates positively with post-traumatic growth in both living and cadaveric kidney transplant recipients.
- Alexithymia correlates negatively with some aspects of post-traumatic growth on kidney recipients from cadaveric donors.
- Resilience can be a predictor for post-traumatic growth in both living and cadaveric kidney transplant recipients.

## Figures and Tables

**Table 1:** Differences in the level of post-traumatic growth between recipients from a living donor and recipients from a cadaveric donor.

PTGI-R	Recipient from a Living Donor M (SD)	Recipient from a Cadaveric Donor M (SD)	t	df	*p*-Value
Overall post-traumatic growth	74 (16)	65 (21)	2.271	86.271	0.03
Changes in self-perception	31 (9)	27 (11)	2.189	89	0.03
Changes in relationships with others	25 (6)	22 (7)	1.593	89	0.12
Greater appreciation of life	13 (2)	11 (4)	2.524	82.274	0.01
Spiritual changes	5 (3)	5 (3)	0.886	89	0.38

M–mean; SD-standard deviation.

**Table 2:** Values of the correlation coefficient of resilience with post-traumatic growth in the recipient from the living donors.

Resilience						
Post-traumatic growth	Factor 1	Factor 2	Factor 3	Factor 4	Factor 5	Overall result
Changes in self-perception	0.255 #	0.424 **	0.524 **	0.651 **	0.582 **	0.552 **
Changes in relationships with others	0.238 #	0.320 *	0.303 *	0.353 *	0.274	0.334 *
Greater appreciation of life	0.170 #	0.265 #	0.395 **	0.506 **	0.319 *	0.299 *
Spiritual changes	0.306 *	−0.015 #	0.215 #	0.093 #	0.057 #	0.123 #
Overall result	0.299 *	0.371 *	0.472 **	0.555 **	0.461 **	0.403 **

Factor 1: Tenacity and determination; Factor 2: Openness for new experiences and sense of humor; Factor 3: Personal competencies of dealing with and tolerating negative emotions; Factor 4: Failure tolerance and approaching life as a challenge; Factor 5: Optimistic approach to life and the ability to mobilize. ** *p* < 0.01; * *p* < 0.05; # *p* = ns.

**Table 3:** Values of the correlation coefficient of resilience with post-traumatic growth in the recipient from the cadaveric donor.

Resilience						
Post-traumatic growth	Factor 1	Factor 2	Factor 3	Factor 4	Factor 5	Overall result
Changes in self-perception	0.586 **	0.381 **	0.417 **	0.423 **	0.387 **	0.448 **
Changes in relationships with others	0.329 *	0.279 #	0.235 #	0.325 *	0.284 #	0.335 *
Greater appreciation of life	0.498 **	0.422 **	0.492 **	0.510 **	0.515 **	0.540 **
Spiritual changes	0.186 #	−0.019 #	0.204 #	−0.029 #	0.109 #	0.125 #
Overall result	0.511 **	0.341 *	0.391 **	0.377 **	0.415 **	0.454 **

Factor 1: Tenacity and determination; Factor 2: Openness for new experiences and sense of humor; Factor 3: Personal competencies of dealing with and tolerating negative emotions; Factor 4: Failure tolerance and approaching life as a challenge; Factor 5: Optimistic approach to life and the ability to mobilize. ** *p* < 0.01; * *p* < 0.05; # *p* = ns.

**Table 4:** The values of the correlation coefficient of alexithymia with post-traumatic growth in a recipient from a living donor.

Resilience				
Post-traumatic growth	Factor 1	Factor 2	Factor 3	Overall result
Changes in self-perception	0.013 #	−0.018 #	−0.127 #	−0.005 #
Changes in relationships with others	0.275 #	0.144 #	0.086 #	0.249 #
Greater appreciation of life	−0.023 #	−0.192 #	−0.114 #	−0.124 #
Spiritual changes	0.265 #	0.265 #	0.447 **	0.442 #
Overall result	0.169 #	0.156 #	0.032 #	0.154 #

Factor 1: Difficulty describing feelings; Factor 2: Difficulty identifying feelings; Factor 3: Outward-orientated thinking. ** *p* < 0.01; Elżbieta Zdankiewicz–Ścigała # *p* = ns.

**Table 5:** The values of the correlation coefficient of alexithymia with post-traumatic growth in a recipient from a cadaveric donor.

Resilience				
Post-traumatic growth	Factor 1	Factor 2	Factor 3	Overall result
Changes in self-perception	−0.387 **	−0.337 *	−0.267 #	−0.312 *
Changes in relationships with others	−0.243 #	−0.273 #	−0.190 #	−0.273 #
Greater appreciation of life	−0.307 *	−0.440 **	−0.222 #	−0.370 *
Spiritual changes	−0.050 #	0.078 #	0.056 #	0.082 #
Overall result	−0.291 *	−0.297 *	−0.206 #	−0.304 *

Factor 1: Difficulty describing feelings; Factor 2: Difficulty identifying feelings; Factor 3: Outward-orientated thinking. ** *p* < 0.01; * *p* < 0.05; # *p* = ns.

**Table 6:** Univariate linear regression analysis predicting posttraumatic growth from resilience for all recipients overall.

	b	SE	β	R^2^	ΔR^2^
Step 1				0.198	0.189
Constant	27.622	9.307			
Resilience	0.557	0.121	0.445 **		

** *p* < 0.01.

**Table 7:** Univariate linear regression analysis predicting posttraumatic growth from resilience for living donor recipients.

	b	SE	β	R^2^	ΔR^2^
Step 1				0.196	0.176
Constant	31.737	13.214			
Resilience	0.529	0.168	0.442 *		

* *p* < 0.05.

**Table 8:** Univariate linear regression analysis predicting posttraumatic growth from resilience for cadaveric donor recipients.

	b	SE	β	R^2^	ΔR^2^
Step 1				0.179	0.16
Constant	26.81	13.311			
Resilience	0.543	0.177	0.423 *		

* *p* < 0.05.

## Data Availability

The data are collected in a database prepared by the research team.
